# Changing the malaria treatment protocol policy in Timor-Leste: an examination of context, process, and actors’ involvement

**DOI:** 10.1186/1478-4505-11-16

**Published:** 2013-05-15

**Authors:** João S Martins, Anthony B Zwi, Karen Hobday, Fernando Bonaparte, Paul M Kelly

**Affiliations:** 1 GlobalHealth@UNSW and School of Public Health and Community Medicine, University of New South Wales, Sydney, Australia; 2 Health, Rights and Development (HEARD@UNSW), School of Social Sciences, University of New South Wales, Sydney, Australia; 3 Ministry of Health, Dili, Timor-Leste; 4 National Centre for Epidemiology & Population Health, College of Medicine, Biology & Environment, Australian National University, Canberra, Australia; 5 Faculdade de Medicina e Ciências da Saúde, Universidade Nacional Timor Lorosae, Avenida Cidade de Lisboa, Dili, Timor-Leste; 6 Population Health Division, ACT Government Health Directorate, Canberra, Australia

**Keywords:** Evidence, Malaria treatment, Policy formulation, Policy process, Timor-Leste, Treatment protocol

## Abstract

In 2007 Timor-Leste, a malaria endemic country, changed its Malaria Treatment Protocol for uncomplicated falciparum malaria from sulphadoxine-pyrimethamine to artemether-lumefantrine. The change in treatment policy was based on the rise in morbidity due to malaria and perception of increasing drug resistance. Despite a lack of nationally available evidence on drug resistance, the Ministry of Health decided to change the protocol. The policy process leading to this change was examined through a qualitative study on how the country developed its revised treatment protocol for malaria. This process involved many actors and was led by the Timor-Leste Ministry of Health and the WHO country office. This paper examines the challenges and opportunities identified during this period of treatment protocol change.

## Key messages

• The process of policy formulation in all settings, including those which are resource-poor, requires considerable attention to identifying, consulting and actively engaging, the range of health system participants that will have an influence on the policy and its implementation.

• In the presence of limited government capacity, expertise from development partners and the non-government sector can add value if drawn into the process and utilized to develop and formulate new policies.

• Strong leadership, at times coupled with potentially unpopular decisions, may be required to drive the policy process and produce desired outcomes in difficult times.

## Introduction

Malaria is endemic in Timor-Leste. Since gaining independence in 2002, the country has changed the Malaria Treatment Protocol (MTP) three times, each associated with a new edition of government malaria treatment guidelines. The first edition was published in 2002, and the second and the third in 2005 and 2007, respectively [1-3].

When Timor-Leste was under Indonesian occupation and administration (1975–1999), the first line treatment for both falciparum and vivax malaria was chloroquine [4]. While chloroquine continued to be recommended in all editions for treating vivax malaria, the treatment for falciparum malaria has changed. The first and the second MTP recommended sulphadoxine-pyrimethamine (SP) for treating uncomplicated falciparum malaria [1,3] while the most recent change recommended artemisinin-based combination therapy (ACT) for this form of malaria

The World Health Organization (WHO) guidelines for malaria treatment in 2006 recommended changing first-line treatment when the total failure proportion of anti-malarial drug exceeds 10% [5]. Countries in South East Asia, such as Indonesia and Thailand which had used SP as the first-line drug for falciparum malaria experienced a rapid increase of parasites resistant to this drug [6]. Consequently, almost all countries in South East Asia replaced SP with ACT in the ensuing years. In 2007, Timor-Leste followed suit and created the third MTP edition which recommended artemether-lumefantrine (AL; *Coartem*^*®*^ - its trade name) as the first-line treatment for uncomplicated falciparum malaria. The revised MTP also included the use of a rapid diagnostic test (RDT) [2]. The revised MTP has been used since 2008 to date [7]. At the launch of the third edition of the MTP in June 2007, the then Minister for Health, Dr Rui Maria de Araujo, stated that the replacement of the existing treatment protocol for malaria was based on the increased reported cases of *P. falciparum* resistance to chloroquine and SP and the increasing use of ACT for treating falciparum malaria in South East Asia region [8]*.*

Changing any government policy requires intense effort; the challenges are not as great when the change is incremental, as in this case, but even in these situations policy change is often contested [9]. It is well accepted that policy change is influenced by the context within which it operates, the actors involved, and the processes through which change occurs [10]. A study of malaria treatment change in two provinces in South Africa, Mpumalanga and Limpopo, found that local data on drug efficacy, official endorsement by the government, gate keeper viewpoints, and political influences together provided the evidence and rationale for the policy shift [11].

Williams et al. noted that rational policy formulation requires: 1) collection of scientifically valid evidence; 2) presentation of evidence in such a way as to attract political attention; 3) consensus-building about the need for a change; 4) ensuring consistency between the new policy with the national drug policy framework; 5) attention to policy implementation; and finally, 6) monitoring and evaluation to inform subsequent policy development [12]. The important point of changing treatment protocol is to avoid the use of inappropriate and in-efficacious anti-malarial drugs as this exacerbates drug resistance which will result in direct and indirect cost to human life, the health system and economic development [13].

The rationale for changing treatment policy due to emerging drug resistance has been well documented [14-16]. However, little is known about the policy process involved in altering a national treatment protocol, an issue explored here. The paper focuses on the reasons for the change, the actors involved, and the challenges encountered.

## Methods

The study examined the process of treatment policy change using a combination of qualitative methods including open-ended interviews with key informants and document reviews. Data were collected between April and July 2008. Ethical clearance was obtained from The University of New South Wales Human Research Ethics Committee (HREC 07231) and national permission granted by the Timor-Leste Ministry of Health (VM-MS/UNSW/07/121).

Twenty-four key informants who had contributed to the development of the protocol were purposively selected. These informants were drawn from the Malaria Working Group, the National Commission for Protocol Finalization (NCPF) and senior Ministry of Health (MoH) officials (former Minister for Health, Vice-Minister, Permanent Secretary, Director of Health Service Delivery, and Director of Health Policy and Planning) who oversaw the protocol formulation process. Eight of the informants worked for the MoH, six for WHO in Timor-Leste, four for NGOs, two from the Timor-Leste Medical Association, and four were private clinicians. Fourteen interviews with Timorese key informants were conducted in Tetum (one of the official languages in Timor-Leste) by the two Timorese researchers (JM and FB). Ten interviews with International agencies were conducted in English by JM. Among those interviewed, nearly two thirds had been actively involved in the formulation team and NCPF, while the others were senior MoH and WHO officials who contributed in other ways to the process.

Open-ended interview questions were designed to elicit data concerning the timing of events, the rationale for changing the protocol, the level of involvement of actors, and their views, experience and influence over the process. Interviews with key informants were conducted face-to-face and normally lasted from half to one hour.

All interviews in both Tetum and English were transcribed; those conducted in Tetum were first translated into English and then checked (AZ and JM). The first author is a native Tetum speaker and consulted the original recording and text as required to clarify meaning. Interview transcripts were imported into NVivo 7 software for coding. Coding was based on the themes derived from text as well as those related to aspects of context, process and actor involvement. Other important themes included the chronology of policy change, the rationale for policy change and the challenges faced during the MTP formulation process.

Documents analysed were relevant to the processes involved in formulation of the MTP and included meeting minutes, reports, circulars, and directive letters issued by the MoH. Data analysis was guided by the grounded theory approach [17,18] and Walt and Gilson’s (1994) policy triangle [10]. Content analysis was also used. Insights from the interviews were triangulated with those of other informants as well as with the document reviews and vice versa.

## Results

### Chronology of malaria treatment policy (MTP 3rd edition) development

In 2005, the Malaria Working Group (MWG), formed by the MoH to provide advice on the implementation of the malaria program, conducted quarterly meetings to assess the progress of malaria programs funded by the Global Fund to Fight AIDS, Malaria and Tuberculosis (Global Fund). Membership was drawn from the MoH, UN agencies and NGOs. In the second half of 2005, meetings held in September and December discussed the possibility of introducing ACT in Timor-Leste. The meetings resulted in two relevant recommendations:

“1. ACT should be introduced in Timor-Leste for effective treatment of uncomplicated *falciparum* malaria especially for high risk groups, pregnant women and children under five;

2. Pilot implementation of ACT should be started in two districts (tentatively, Lautem and Bobonaro) after strengthening of laboratory services, development of ACT using guidelines, training of district health staff and certainty of long-term availability of ACT is established” [19].

The MWG sought advice from the Minister for Health regarding the process of modifying the MTP. Since a study on drug efficacy was deemed not feasible (discussed further below), the Minister gave instructions to change the malaria treatment protocol on the basis of evidence from outside Timor-Leste. The MWG commenced researching and compiling relevant evidence. This was followed by the drafting of the MTP by the MoH Department of Communicable Disease Control (CDC) and WHO between February 2006 and May 2007.

In May 2007, the MoH established the NCPF with 16 members from the MWG, the Cuban Medical Brigade (which for a number of years has provided support in Timor-Leste), NGOs, health professional associations and the private sector. The NCPF was chaired by a senior medical doctor from a private clinic and the head of the CDC Department, MoH. The role of the NCPF was to provide technical inputs, finalise the revised MTP, approve the final draft, and ensure socialisation (introductory training and dissemination) and implementation of the protocol. The NCPF held its first meeting on the 3^rd^ of May 2007 followed by a three-day workshop. An agreed final draft was available by the 16^th^ of May 2007.

The revised MTP was officially launched by the Minister for Health on 12 June 2007. Translation into Tetum (to ensure availability to local Timorese health care staff) and Spanish (to inform treatment by personnel associated with the Cuban Medical Brigade) took place between July and September 2007. The socialisation of the new MTP took place between October and December 2007 [7].

In January 2008, the Permanent Secretary of the MoH issued a directive letter to all 13 District Health Services and all hospitals in the country instructing them to implement the revised MTP. A similar letter was issued again in March 2008 to the country’s District Health Offices to implement this MTP by the newly appointed Director General who replaced the Permanent Secretary. A study aiming at evaluating the implementation of this new MTP has been published [7].

### The actors, context and process

**Figure 1:**
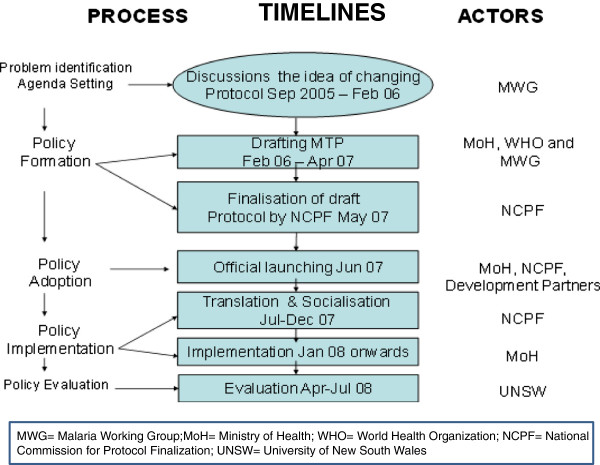
Process, timelines and actors.

Figure 1 illustrates the processes, timelines and the actors involved in the MTP formulation process, using Anderson’s model of the policy cycle [20].

#### Actors

The main factors involved in the formulation of the MTP included the MoH, WHO, MWG and NCPF. The MoH assigned its own CDC Department, WHO and the MWG to jointly initiate drafting the MTP. Within a few months the draft was produced and shared with the NCPF.

Actors’ participation in the development of the MTP included providing technical advice on protocol development, providing clinical expertise and opinion, reviewing and editing the content of the protocol, providing translation and disseminating the MTP (socialisation) (Table 1).

**Table 1:** Summary of actors’ roles and their contributions to the MTP development

**Actors/Organisations**	**Main responsibility**	**Contribution to MTP development**
Ministry of Health	Develop malaria policies, coordinate and implement the malaria control program	Draft the protocol, lead process and finalisation of the protocol.
World Health Organization country office in Timor-Leste	Provide technical assistance needed by the MoH including policy development	Draft the protocol, search for evidence from the literature and seek advice from malaria experts in other countries. Assist the MoH in protocol formulation, completion and official approval.
Non-government organizations (HNI, CARE International, CRS, HAI)	Implement malaria control program particularly in health promotion and prevention activities	Some clinicians working with the NGOs provide technical expertise in relation to drug dosages and the application of the protocol in the IMCI program.
Bilateral – USAID through its agency TAIS	Implementing Child Survival Programs – mainly involved in ITN distribution	Contribute inputs to the finalisation of the draft protocol.
		USAID offered to conduct SP efficacy study, but this was not taken forward.
Cuban Medical Brigade	Provide curative service but also health promotion and prevention activities.	Contribute to the finalisation of the draft protocol and Spanish translation.
Medical Association	Provide curative service, health promotion and prevention activities.	Contribute to the finalisation of the draft protocol, translation and socialisation.
Private Clinics	Curative service	One private clinic had used Artemisinin-based combination therapy prior to the official adoption by the MoH.
National Commission for Protocol Finalisation	The NCPF members drawn from the various organisations	Finalise the draft protocol, approve the MTP text, translate the MTP into Tetum and Spanish, and conduct socialisation.
The Global Fund to fight AIDS, Tuberculosis and Malaria	Provide funding for the Malaria Control Program	The Global Fund created the MWG. The MWG was instrumental to the proposed idea of changing MTP for *P. falciparum* from SP to AL. Assist with the literature review to find evidence for changing the protocol and assist with drafting the protocol.
External expert	WHO SEARO had expertise in guideline development and RBM strategies	Provide advice on the protocol and development of the treatment guidelines including the formulation of drug dosages.
WHO-Regional Office and Mahidol University	Mahidol University provided pharmacological expertise	

AL: Artemether-Lumefantrine; CRS: Catholic Relief Services; HAI: Health Alliance International; HNI: HealthNet International; MTP: Malaria Treatment Protocol; MWG: Malaria Working Group; NCPF: National Commission for Protocol Finalisation; RBM: Roll Back Malaria; SP: Sulphadoxine-Pyrimethamine; TAIS: Timor-Leste Assistência Integrado Saúde; USAID: United States Agency for International Development; WHO SEARO: World Health Organization South East Asia Regional Office.

#### Context

The existing collaboration between the MoH and the NGOs involved in the Global Fund provided an enabling environment for revising the MTP [21]. Through the MWG, the actors were already familiar with each other and this facilitated cooperation and collaboration on both programmatic and policy issues.

The absence of a human research ethics committee, lack of funding and national experts in malaria research influenced the MoH’s decision to consider it unfeasible to undertake a drug efficacy study but instead to change the MTP based on available international evidence.

The external context was characterised by rising evidence of SP resistance in neighbouring countries (particularly Thailand and Indonesia) [15,16,22]. Another stimulus for changing the protocol was the political decision at the Ministerial Meeting in Dhaka, Bangladesh, in 2006 to adopt ACT as the standard treatment for the *P. falciparum* infections in South-East Asia Region [23,24].

However, there were also inhibiting factors which affected the protocol formulation process. A major part of the protocol development took place in 2006 and 2007 at a time of major political instability in the country, particularly in the capital, Dili [25,26]. This insecurity affected participation in this process and caused a longer than anticipated MTP formulation process.

#### Process

This section examines the lead-up to the MTP change. It examines the rationale for, and initiation of, the change, and considers the role of the NCPF in particular.

##### How and where the change began

As already indicated, the idea for changing the MTP came from the MWG, taking account of the high malaria burden in the country in which the annual clinical malaria incidence showed an unstable rate of 236.1 per 1,000 population in 2004 and 212.4 per 1,000 population in 2007 [21]. Apart from this, a desire to improve diagnosis was also part of the rationale. In 2007 it was reported that the correct diagnosis of malaria through the use of microscopy was as low as 28 to 40% across the country, with laboratory diagnosis occurring primarily in the few hospitals and Community Health Centers (CHCs) located in district capitals.This meant that the majority of the population who sought treatment in health facilities in remote areas such as Health Posts and Mobile Clinics, and even some CHCs, did not have access to proper diagnosis. In addition, the reagents needed for microscopic examination of malaria parasite were not readily available adding further difficulties [27]. This resulted in encouraging the MoH and the WHO to go ahead with the idea of changing the treatment protocol and to begin the process of altering the policy on malaria treatment in the country.

Timor-Leste Assistência Integrado Saúde (TAIS), a USAID funded Agency, to the Minister of Health to ask a possibility of conducting an efficacy study on SP, but it was felt not feasible at that time due to the lack of a functioning human research ethics committee:

*“…the option discussed at that time was [to conduct a] therapeutic efficacy study to find out the efficacy of SP, but the conditions at that time seemed to be difficult to undertake this study due to various reasons like there was no ethics committee and others. At that time [the] Minister gave a solution [that] if there was already evidence available then we [should] just change it directly from SP to ACT”* (Malaria Officer, MoH).

The MoH believed that it was not necessary to replicate studies that had been conducted in other countries which were likely to produce similar results and thus represented an unnecessary waste of time and resources.

*“One of my concerns was the proliferation of studies without any significant need. What I mean by any significant need is if there is evidence out there about effectiveness or ineffectiveness of X, Y or Z medicine, why should we do it in our country again, why should we lose time going through studies”* (Minister for Health 2001–2007).

The decision was made to change to ACT based on international experience and advice, with efforts geared toward collecting the published evidence which would underpin this decision. This was followed by the drafting of the MTP initiated by the CDC-MoH, the WHO and the MWG. Expert opinions from the WHO regional office and Mahidol University in Thailand were also sought.

*“We drafted it together…major [part of the] protocol development was in consultation not only with the WHO, it was consulted with some scientists, particularly from Thailand”* (WHO country representative 2000–2007).

The draft was then presented back to the Minister and was approved. Plans for introducing the protocol to health workers and the training for implementation were then developed.

Actors involved in the formulation team expressed their views about the policy process as reflected here. According to them, this was the first time in Timor-Leste that the formulation of a treatment protocol was collectively undertaken with the involvement of a wide range of actors. It was suggested that other protocols should undergo a similar process.

*“It’s good because it’s not only one person’s work, it is team work. We compare it with other guidelines and protocol [developed], this is the first time getting Timorese doctors involved”* (WHO staff and NCPF member).

Most of them agreed with the change, warning that the cost to human life and to the economy would otherwise be substantial.

*“I think it was right time to change…not changing it might have [led to] more cases of malaria, more deaths, work force and economic loss for the country”* (Paediatrician, NGO senior staff and NCPF member).

Aside from the positive views about the MTP policy process, there were also concerns expressed that the MTP process was a top down process and the involvement of a technical committee was seen as an effort to legitimise the process.

*“[It was] a top down approach, a technical committee was used more as the process of validation than technical debate. Limited knowledge, skills and expertise of committee members might have created this scenario”* (NGO worker and NCPF member).

The private sector appreciated the opportunity to participate but lamented that they had not been fully engaged in the policy process.

*“It seems it is very difficult for the government to involve the private sector, even if they do, when the process is nearly complete, so this is not an involvement, but an introduction”* (Senior medical practitioner in Dili).

Despite these concerns, it was agreed that it was best for the country to change the MTP.

##### Rationale for changing the protocol according to the key informants involved in the protocol formulation

The most important reasons stated for the change are reflected in the quotes below and included perceived SP resistance, limited access to proper diagnosis, and the rising incidence of malaria (Table 2).

*“First of all, this was based on the existing high speed of rising drug resistance to SP which was documented in Indonesia…second is WHO’s common understanding of the unique opportunity to introduce drugs which Timorese really required…that was the reason behind this”* (WHO country representative 2000–2007).

**Table 2:** Reasons for changing the treatment protocol according to participants involved in MTP development

**Participants**	**Reasons stated**	**Frequency**	**Percentage**
n = 24	Perceived SP resistance	17	71%
	No access to proper diagnosis and treatment	8	33%
	Rising malaria incidence	6	25%
	Health Minister’s request to change	4	17%
	Fulfilling political commitment at regional level	2	8%
	Losing faith in SP	2	8%
	The desire to standardise malaria treatment	1	4%
	ACT not yet resistant and price drop	1	4%

Note: more than one reason mentioned by informants.

The issue of perceived resistance to SP was reinforced by clinical experience:

*“I can tell you as a clinician, seeing patients all the time, maybe as much as four years ago I started to see when we gave Fansidar [SP] certain patients are not getting better and [in years] before almost everyone was getting better, so I started to lose faith in Fansidar a long time ago”* (Senior medical practitioner in Dili).

Policy makers saw that the change of the MTP in Timor-Leste was complemented by efforts to improve diagnostic practices from a symptomatic approach to a more reliable diagnosis as well as improving treatment practices.

*“I think the main concern at the time was that malaria treatment was not widely accessible by the community in terms of appropriate diagnosis and appropriate treatment. One of the issues was appropriate diagnosis; it was found that microscope examination [to detect the malaria parasites in the blood] was not provided widely throughout the country.”* (Minister for Health 2001–2007).

The intention to provide effective treatment was also related to ethical issues; the belief that it would have been unethical to continue treating patients with inefficacious drugs. In addition, treating patients with efficacious drugs is more economical as it prevents complications which could lead to further costs.

##### The NCPF and its role

As explained earlier, the NCPF’s role was to review and agree on the final draft of the MTP before official approval was granted. In reviewing and examining the content of this draft protocol, the NCPF went through a meticulous process to finalise the draft.

*“First we looked at the general things, and then we gave them the draft and asked them to make comments. They could give their comments through email…our task was to receive the inputs, when the inputs came, we analysed them together. After this, we conducted meetings twice per week, again we also looked through them page by page, we looked at the dosages”* (WHO staff and NCPF member).

The work of the NCPF culminated with the adoption of the final draft of the revised MTP on 16 May 2007. Key members involved in developing the protocol acknowledged the contribution of the NCPF. Although the draft protocol had been prepared, the NCPF improved the content of the existing draft.

*“Because this draft was already there, [NCPF] contributions in terms of technical matters…they enriched the draft”* (Head of CDC and co-chairman of the NCPF).

### Challenges during the formulation of the new protocol

Challenges during the development of the revised MTP included difficulties in managing people in the working group, resistance from some health workers, divergence of implementation strategy, language difficulties, and uncertainty about the government’s capacity to implement the change.

The formulation process of the protocol involved many actors and some NCPF members experienced difficulties in managing people with diverse experiences and backgrounds.

*“It took a bit longer because to make this protocol, the discussion was a little bit complicated…there were many ideas that emerged at the time of developing this protocol, some people had experience (working) in Africa, they would say their experience”* (MoH Malaria Officer).

Communication was also complex in terms of the difficulty in reaching the NCPF members by telephone. The language used to draft the protocol was English and the reading materials were also in English.

The lack of data on the resistance status of SP to *P. falciparum* available within Timor-Leste left some NCPF members still believing that SP was effective.

*“My observation [is that] SP is still sensitive [to P. falciparum], though there were one or two cases of resistance, we gave second line treatment. We observed this based on clinical basis”* (A doctor and NCPF member).

Some NCPF members from NGOs and private sector pushed for the inclusion of community health volunteers in providing diagnosis and treatment with an argument that people in remote areas would continue to have no access to early treatment if the RDT examination and AL could only be delivered by health workers.

However, the MoH maintained its position to only allow trained health workers to conduct diagnosis and treatments. Others raised concerns over the weak drug supply system and the readiness of the health sector to embrace and ultimately implement the change.

## Discussion

The increase of malaria cases, the need to improve diagnosis and treatment, and the perceived rise in SP resistance prompted Timor-Leste to change its treatment protocol for malaria.

As Walt and Gilson (1994) have argued, many policies in developing countries have failed to reach their final objective because the policy process paid more attention to content while giving little or no attention to context, process and actors [10]. The formulation of this treatment protocol demonstrates that while the content, that is changing of a drug regimen, was required, the process needed to devote attention to the actors involved and the context in which policy change and implementation was to occur.

Three important factors drove the process of changing the MTP. Firstly, there was a demand from people working on the ground to have the protocol changed; these included the MWG and a number of other clinicians. Secondly, the role of the technical departments, in this case, the CDC Department within the MoH and WHO, which reinforced the idea of changing the MTP from the first group and proactively approached the Minister to suggest the change. This second group also actively sought evidence on drug (SP) resistance from neighbouring countries [15,16,22,28] to justify their argument. The active policy process pursued by the second group was further strengthened with the appointment of the NCPF which expanded the participation of stakeholders with a mandate to approve the final draft of the protocol before official approval from the Minister for Health. Thirdly, the Minister’s role was key. His leadership enabled change in the MTP without a local efficacy study being conducted. The Minister’s decision drove forward the policy process.

Most informants indicated that even if the decision had been made to conduct a study, in the end, the result would have been to recommend changing the then SP regimen to a more potent anti-malarial drug, ACT, as the preferable option. Many would have liked local data to support the change, but the scarcity of resources available in the country made this option not feasible. The decision made by the Minister has been viewed as “undermining” the importance of locally generated evidence on drug resistance. However, it can also be argued that the Minister for Health had sufficient confidence and leadership to argue there was “good enough” (albeit incomplete) evidence to avoid delays and make the required changes. Zambia went through a similar situation when it changed its treatment to AL; there too the direct intervention of the Minister sped up the policy process of developing a treatment protocol for malaria [29]. This policy process fits with the Kingdon model of three streams: problems, politics and policy. The three streams may each invite policy change, but this might have taken place more slowly or not at all. In conditions where more than one stream is present, policy change tends to speed up [30]. In the context of Timor-Leste malaria policy, we can equate the rising cases of malaria and MWG and clinicians who advocated for a change of treatment as problem streams. The CDC Department and WHO saw the case (problem) presented by the MWG to change the treatment guidelines and argued for a change in policy (policy stream), while the Minister’s decision reinforced the politics stream – including a view that if the end result was to be the same then why not initiate the change as soon as possible.

Evidence from the South-East Asia region, particularly Indonesia [22], Thailand, Myanmar, Laos and Cambodia [16,31-33] supported the change. Timor-Leste was not alone in changing treatment in the absence of local evidence, several other countries like Peru, Kenya, Tanzania and Zambia, also changed their treatment protocols largely on the basis of international evidence [29,34]. The Ministerial Meeting in Bangladesh in 2006, which approved ACT as the standard for treating falciparum malaria for all SEARO countries, was also influential [23]. A Timor-based study by Almeida et al. published after the approval of the MTP subsequently confirmed high rates of resistance of SP to *P. falcifarum* (82.3%) [35], underpinning the Minister’s belief at the time in the case for policy change.

It was well recognised that the local actors did not have much and/or any experience in policy development and this was certainly a challenge. However, the limited experience of local actors in policy development was offset by drawing on other locally available or sourced expertise from the WHO and participants in the MWG and NCPF.

The protocol was developed in 2006–2007 which coincided with a period of conflict and instability, particularly in Dili [25,26]. Key meetings, discussions and workshops are, and in this case were, held in Dili, the capital city. Conflicts have the potential to reduce the capacity in policy making, planning and implementation [36,37], but apart from delaying some meetings, this did not inhibit the work of the actors involved in the development of the MTP. Indeed, the conflict in 2006 strengthened the resolve, and in some senses, the capacity of the MoH to deliver health services to the population. A commitment of the MoH leadership, plus the availability of extra resources to address emergent needs, helped ensure that most health needs of the displaced population and general population were met [25,26]. This demonstrated the importance of leadership in maintaining the functions of institutions. For example, the MoH was the lead health institution during the political instability and seized opportunities to introduce necessary changes for longer term benefit. The new MTP was a concrete example of seizing opportunities in a political instability or crisis to promote change. Therefore, it confirms that whilst political crisis may bring violence and destruction of people’s lives, there may also be “windows of opportunity” to introduce longer term and positive change [37].

The study highlights the importance of interactions between actors, content and context in policy development processes, and also reinforces the importance of leadership, presented here as the ability to make key decisions based on country needs and available information and evidence, intelligently analysed.

Timor-Leste offers valuable lessons to other countries facing similar challenges. In resource-poor countries, developing good policies or changing unworkable (out-dated) policies can occur when governments can skilfully mobilise expertise from the non-government sector and work collaboratively with them to drive the desired changes. The formulation of the new MTP in Timor-Leste is one such example. Equally important was bringing together actors from different organisations into the protocol development process: this served as a venue where actors with little or no experience could develop skills in interacting with processes concerned with developing treatment guidelines and protocols. These reflect also the importance of supporting the development of professional and institutional capacity.

## Conclusion

The development of the revised MTP coincided with a difficult period of instability in Timor-Leste. The rationale behind the change was driven by the increase of malaria cases, the need to improve diagnosis and treatment, and the perception of increasing drug resistance. The absence of local evidence did not inhibit the process given the leadership by the Minister for Health and his staff who argued there was enough evidence, in a variety of forms, from the country and region to promote the policy change. The case and evidence-base for policy change was good enough and did not need to await final scientific “proof”.

The policy change was developed in a cooperative nature involving interactions between a range of local, national and expatriate individuals and agencies, within and outside of government. While this may have slowed aspects of the process, it bore fruit during policy implementation given the sense of ownership by many of those involved. Some weaknesses remained and have been described elsewhere [7]. This study also demonstrates that while political crises bring violence, destruction and disruption of people’s lives and livelihoods, quality leadership and development partner support can, even in these situations, open and see through “windows of opportunity” to introduce longer term policy and systems change.

## Abbreviations

ACT: Artemisinin-based combination therapy; AL: Artemether-lumefantrine; CDC: Communicable disease control; MoH: Ministry of Health; MTP: Malaria treatment protocol; MWG: Malaria working group; NCPF: National Commission for Protocol Finalization; RDT: Rapid diagnostic test; SP: Sulphadoxine-pyrimethamine (SP).

## Competing interests

The authors declare that they have no competing interests.

## Authors’ contributions

JM is a PhD graduate at the University of New South Wales. This study was part of his PhD thesis. JM was involved in conceptualising this study, conducting data collection, data analysis, writing up the first draft of this paper and subsequently contributed to all stages of this paper until finalisation. AZ was supervisor for JM’s PhD studies of which this paper is one component. AZ contributed to conceptualising this research and data analysis, and contributed to writing up and finalising this paper. KH contributed to data collection, study design and write-up. She also contributed to shaping the content and editorial presentation of this paper. FB contributed to data collection, study design and was involved in drafting the earlier version of this paper. FB provided views as an insider and outsider to this paper. As an insider, he was co-chairman of the National Committee for Protocol Finalisation and as an outsider, he was involved in this study as a researcher. FB passed away without seeing the final draft. PK was co-supervisor for JM’s PhD studies. PK was involved in study design, data analysis and presentation, and all aspects of the write-up for publication. All authors (except the late FB) read and approved the final manuscript above.
